# Supplementary material for: Real World Analysis of Quality of Life and Toxicity in Cancer Patients Treated with Hyperthermia Combined with Radio(chemo)therapy
Source: Cancers (Basel). 2023 Feb 15;15(4):1241. doi: 10.3390/cancers15041241 (PMC9954584; doi:10.3390/cancers15041241)
Supplement: Supplementary file 1 [file cancers-15-01241-s001.zip › cancers-2189831-supplementary.pdf]

# Supplementary Material

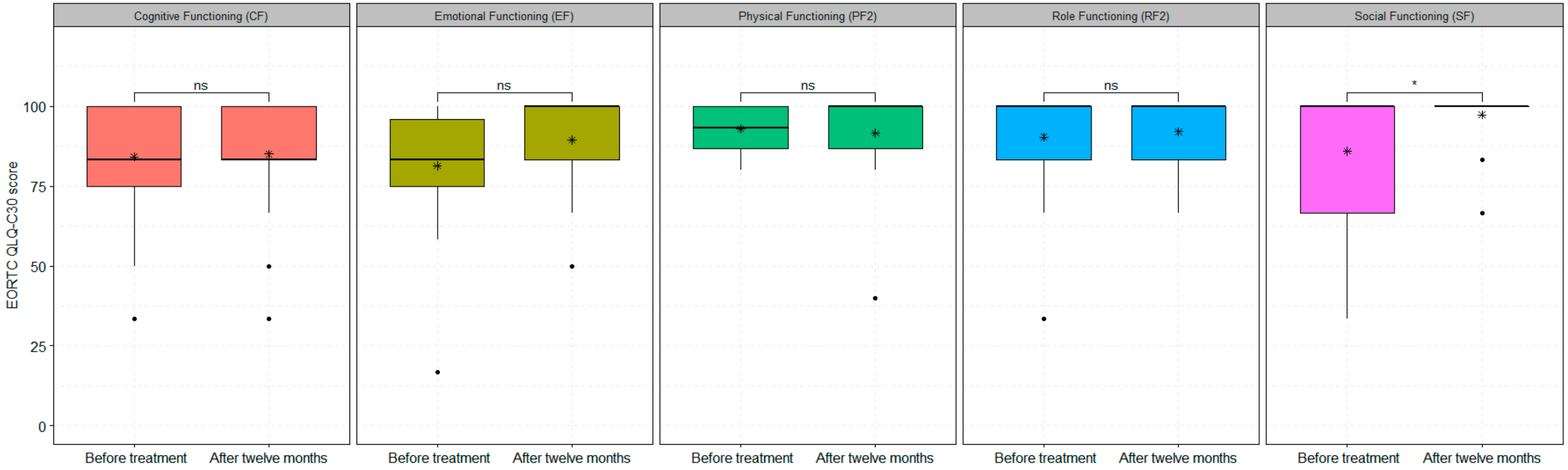


**Figure S1**. The functional scale scores of 19 patients treated with curative RCT and superficial HT before and twelve months after treatment for: CF (84.21 ± 19.62 vs 85.08 ± 19.16, p-value=0.91), EF (81.43 ± 20.26 vs 89.47 ± 14.39, p-value=0.13), PF2 (92.98 ± 8.15 vs 91.57 ± 14.7, p-value=0.74), RF2 (90.35 ± 16.95 vs 92.10 ± 12.87, p- value=0.83), SF (85.96 ± 20.98 vs 97.36 ± 8.35, p-value=0.048). The asterisks (*) represent the mean values; ns: non-significant with p-value > 0.05; *: significant with p-value < 0.05;

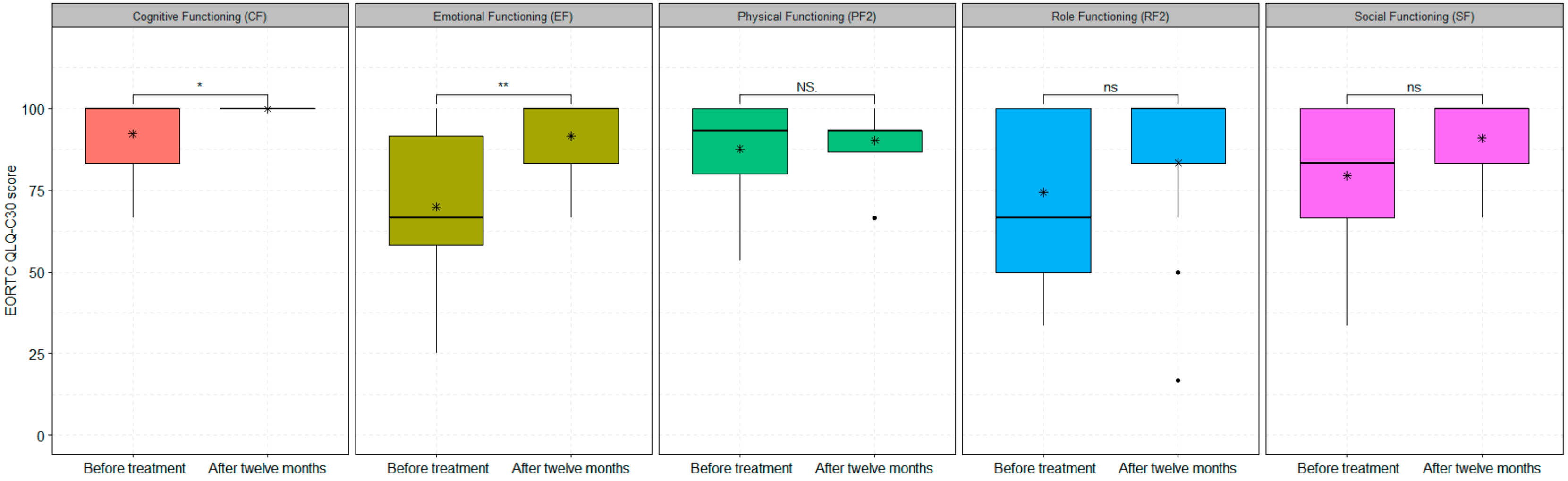


**Figure S2**. The functional scale scores of 13 patients treated with curative RCT and deep regional HT before and twelve months after treatment for: CF (92.30 ± 11.01 vs 100 ± 0.0 , p= 0.017), EF (69.87 ± 23.20 vs 91.66 ± 12.72, p= 0.008), PF2 (87.69 ± 15.11 vs 90.25 ± 8.86, p-value=1), RF2 (74.35± 24.16 vs 83.33 ± 25.45,p-value=0.3), SF (79.48 ± 20.58 vs 91.02 ± 14.61,p-value=0.1). The asterisks (*) represent the mean values; NS: p-value =1; ns: non-significant with p-value > 0.05; *: significant with p-value < 0.05; **: significant with p-value < 0.01;

**Table S1.** A comparison of functional scale items of patients treated with curative RCT in combination with HT 12 months after treatment and the EORTC general population.

| Functional scales items | | Mean | SD | Mean ref. | SD ref. | p-value |
|---|---|---|---|---|---|---|
| Cognitive Functioning | CF | 91.1 | 16.4 | 82.6 | 21.9 | 0.064 |
| Emotional Functioning | EF | 90.4 | 13.6 | 71.4 | 24.2 | 0.0003 |
| Physical Functioning | PF2 | 91.0 | 12.5 | 76.7 | 23.2 | 0.0034 |
| Role Functioning | RF2 | 88.5 | 19.1 | 70.5 | 32.8 | 0.009 |
| Social functioning | SF | 94.8 | 11.5 | 75.0 | 29.1 | 0.0009 |

**Table S2.** A comparison of functional scales items of patients treated with palliative RCT in combination with HT three months after treatment and the EORTC general population.

| Functional scales items | | Mean | SD | Mean ref. | SD ref. | p-value |
|---|---|---|---|---|---|---|
| Cognitive Functioning | CF | 85.1 | 16.3 | 82.6 | 21.9 | 0.723 |
| Emotional Functioning | EF | 78.1 | 19.4 | 71.4 | 24.2 | 0.177 |
| Physical Functioning | PF2 | 77.2 | 23.7 | 76.7 | 23.2 | 0.815 |
| Role Functioning | RF2 | 72.8 | 31.8 | 70.5 | 32.8 | 0.588 |
| Social functioning | SF | 72.8 | 28.6 | 75.0 | 29.1 | 1.00 |

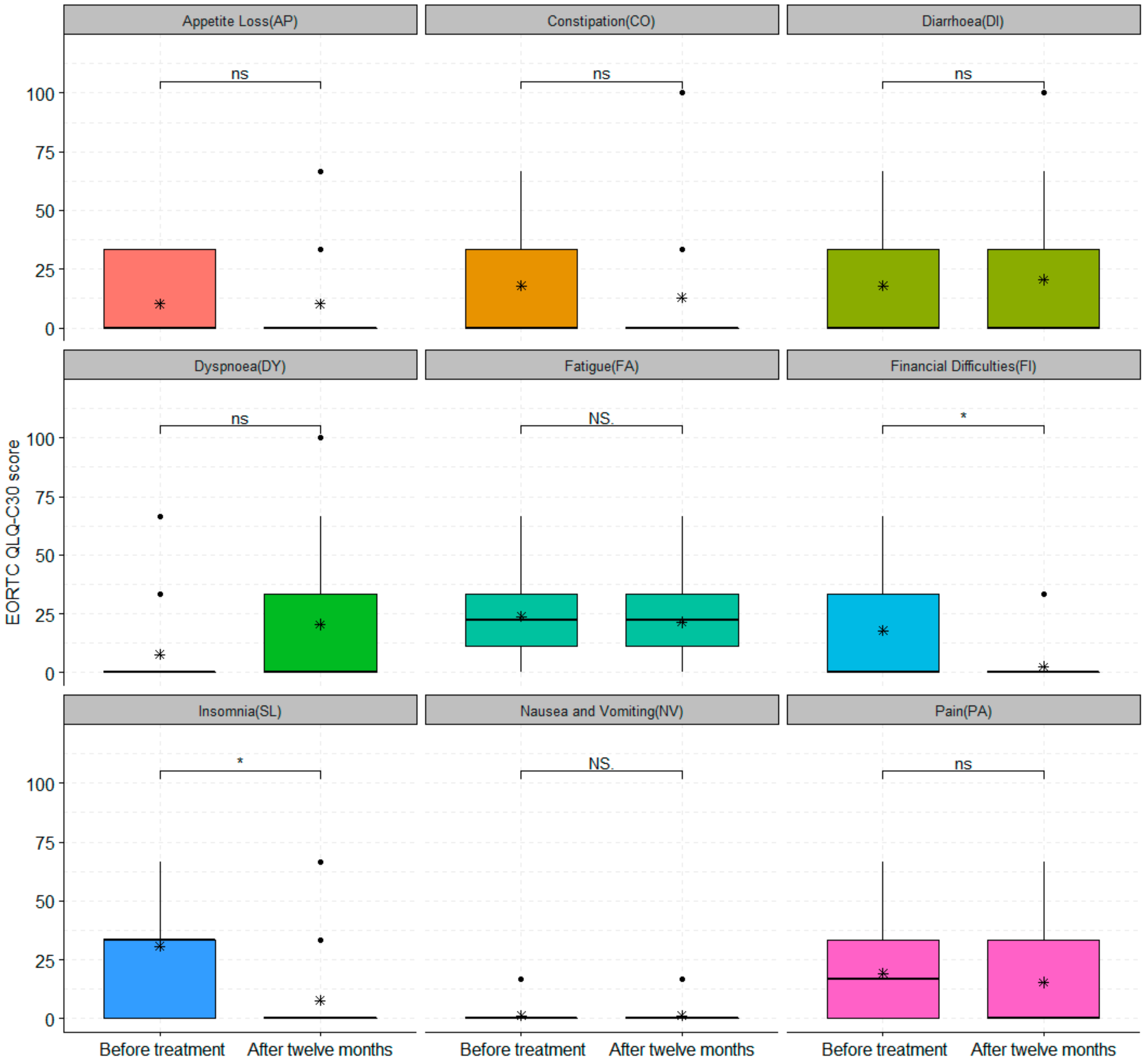


**Figure S3.** The symptom scale scores of 13 patients treated with curative RCT and deep regional HT before and after treatment for: AP (10.25 ± 16.01 vs 10.25 ± 21.01, p-value=0.79), CO (17.94 ± 22 vs 12.82 ± 28.99, p-value=0.3), DI (17.94±22.01 vs 20.51 ± 32.02, p-value=0.91), DY (7.69 ± 19.97 vs 20.51 ± 32.02, p-value=0.21), FA (23.93 ± 22.61 vs 21.36 ± 18.40, p-value=1.00), FI (17.94 ±22 vs 2.56 ± 9.24, p-value = 0.031), NV (1.28± 4.62 vs 1.28 ± 4.62, p-value=1.00), PA (19.23 ± 20.23 vs 15.38± 23.03, p-value=0.47), SL (30.76 ± 25.31 vs 7.69 ± 19.97, p-value=0.012); The asterisks (*) represent the mean values; NS: p-value =1; ns: non-significant with p-value > 0.05; *: significant with p-value < 0.05;

**Table S3.** A comparison of symptom scale items of patients treated with curative RCT in combination with HT twelve months after treatment and the EORTC general population.

| Symptom Scales items | | Mean | SD | Mean ref. | SD ref. | p-value |
|---|---|---|---|---|---|---|
| Appetite loss | AP | 4.2 | 14.0 | 21.1 | 31.9 | 0.006 |
| Constipation | CO | 4.2 | 14 | 17.5 | 28.4 | 0.017 |
| Diarrhoea | DI | 10.4 | 23.1 | 9 | 20.3 | 0.791 |
| Dyspnoea | DY | 14.6 | 26.6 | 21 | 28.4 | 0.344 |
| Fatigue | FA | 19.8 | 19.5 | 34.6 | 27.8 | 0.013 |
| Financial difficulties | FI | 2.1 | 8.2 | 16.3 | 28.1 | 0.006 |
| Nausea and vomiting | NV | 0.5 | 2.9 | 9.1 | 19 | 0.012 |
| Pain | PA | 15.1 | 20.8 | 27 | 29.9 | 0.062 |
| Insomnia | SL | 12.5 | 20.3 | 28.9 | 31.9 | 0.009 |

**Table S4.** A comparison of functional scales items of patients treated with palliative RCT in combination with HT three months after treatment and the EORTC general population.

| Symptom Scales items | | Mean | SD | Mean ref. | SD ref. | p-value |
|---|---|---|---|---|---|---|
| Appetite loss | AP | 23.3 | 32.9 | 21.1 | 31.9 | 0.783 |
| Constipation | CO | 16.6 | 28.7 | 17.5 | 28.4 | 0.907 |
| Diarrhoea | DI | 17.7 | 29.9 | 9 | 20.3 | 0.180 |
| Dyspnoea | DY | 18.8 | 24.2 | 21 | 28.4 | 0.747 |
| Fatigue | FA | 33.3 | 24.4 | 34.6 | 27.8 | 0.845 |
| Financial difficulties | FI | 10 | 23.4 | 16.3 | 28.1 | 0.327 |
| Nausea and vomiting | NV | 13.3 | 23.7 | 9.1 | 19 | 0.435 |
| Pain | PA | 24.4 | 25.0 | 27 | 29.9 | 0.708 |
| Insomnia | SL | 24.4 | 31.4 | 28.9 | 31.9 | 0.559 |
